# A classification method of gastric cancer subtype based on residual graph convolution network

**DOI:** 10.3389/fgene.2022.1090394

**Published:** 2023-01-04

**Authors:** Can Liu, Yuchen Duan, Qingqing Zhou, Yongkang Wang, Yong Gao, Hongxing Kan, Jili Hu

**Affiliations:** ^1^ School of Medical Informatics Engineering, Anhui University of Chinese Medicine, Hefei, Anhui, China; ^2^ Anhui Computer Application Research Institute of Chinese Medicine, China Academy of Chinese Medical Sciences, Hefei, Anhui, China

**Keywords:** multi-omics, autoencoder, patient similarity network, residual graph convolutional network, classification

## Abstract

**Background:** Clinical diagnosis and treatment of tumors are greatly complicated by their heterogeneity, and the subtype classification of cancer frequently plays a significant role in the subsequent treatment of tumors. Presently, the majority of studies rely far too heavily on gene expression data, omitting the enormous power of multi-omics fusion data and the potential for patient similarities.

**Method:** In this study, we created a gastric cancer subtype classification model called RRGCN based on residual graph convolutional network (GCN) using multi-omics fusion data and patient similarity network. Given the multi-omics data’s high dimensionality, we built an artificial neural network Autoencoder (AE) to reduce the dimensionality of the data and extract hidden layer features. The model is then built using the feature data. In addition, we computed the correlation between patients using the Pearson correlation coefficient, and this relationship between patients forms the edge of the graph structure. Four graph convolutional network layers and two residual networks with skip connections make up RRGCN, which reduces the amount of information lost during transmission between layers and prevents model degradation.

**Results:** The results show that RRGCN significantly outperforms other classification methods with an accuracy as high as 0.87 when compared to four other traditional machine learning methods and deep learning models.

**Conclusion:** In terms of subtype classification, RRGCN excels in all areas and has the potential to offer fresh perspectives on disease mechanisms and disease progression. It has the potential to be used for a broader range of disorders and to aid in clinical diagnosis.

## 1 Introduction

Gastric cancer (GC) is a highly aggressive cancer with significant heterogeneity in terms of cell types, states, and subpopulation distribution in the immune microenvironment (Shao et al., 2021; Kim et al., 2022). According to the epidemiological survey (Ferlay et al., 2021), the incidence of GC is the fifth highest among tumor diseases worldwide, and the mortality rate is the third highest among tumor deaths (Wang et al., 2021; Dong et al., 2021). Studies have shown that several variables, including genetics, the immune system, lifestyle choices, and psychological factors, can affect the development and occurrence of tumors (Shin et al., 2022). Multiple pathological processes at various levels and dimensions, including the genome, transcriptome, and proteome, are involved in complex diseases like cancer (Menyhárt and Győrffy, 2021).

With the advancement of high-throughput sequencing and omics technology, researchers progressively understood the limits of employing a single omics (Sun et al., 2019; Jia et al., 2022). To better understand the essence of the disease, it is required to undertake a joint analysis of various types of data, get more comprehensive information, construct a perfect body regulatory network, and thoroughly investigate the regulation and causal relationships between molecules (Tao et al., 2020). Consequently, one of the areas of research that is now quite active is the integration of multi-omics data for cancer subtyping (Lindskrog et al., 2021; Sivadas et al., 2022). The biological information contained in multi-omics data is critical for disease diagnosis and treatment. However, due to its huge scale, high dimension, high noise, and strong heterogeneity, data is difficult to handle and analyze, posing significant obstacles to cancer typing (Duan et al., 2021; Picard et al., 2021).

The Graph Convolutional Network (GCN) (Kipf and Welling, 2017) is a convolutional neural network that was built in recent years that can directly act on graphs and use their structural information, and it is gaining popularity in the field of bioinformatics (Zhang et al., 2021). It can identify unlabeled nodes and categorize them using both the node’s feature vector and network topology data (Li et al., 2022).

Kim et al., (2021) proposes an analytical framework named DrugGCN based on gene expression data for predicting drug responses using graph convolutional networks (GCNs). Baul et al., (2022) offers omicsGAT, a graph attention network (GAT) model that blends graph learning with attention processes for cancer subtype identification based on RNA-seq data. By allocating various attention coefficients to nearby samples, the multi-head attention mechanism can more successfully protect the connection between them. However, such experimental results are neither applicable nor interpretable when only one set of omic data is considered. According to studies (Sun and Hu, 2016; Xu et al., 2019), different forms of data have complementarities, and multi-omics can fuse the rich information in each type of data to facilitate categorization. Li et al., (2022) developed a multi-omics ensemble model, MoGCN, with two-layer graph convolutional networks for the classification and analysis of cancer subtypes. Ramirez et al., (2020) constructed a graph convolutional neural network for classifying tumor and non-tumor samples based on unstructured gene expression data. Unfortunately, as depth increases, graph convolutional networks suffer from vanishing gradients and over-smoothing, which significantly reduces model accuracy. Zhang et al., (2022) proposes a new method for detecting liver cancer using a fusion similarity network, denoising autoencoder, and dense graph convolutional neural network. Liang et al., (2021) proposed a Consensus Guided Graph Autoencoder (CGGA) to identify cancer subtypes and bring fresh insights into the treatment of patients with diverse subtypes. Wang et al., (2021) introduces a unique multi-omics integrative approach called the Multi-Omics Graph Convolutional Networks (MOGONET), which is utilized for biomedical classification and can find key biomarkers from various omics data sources. Finally, Dai et al., (2021) combined GCN with a residual network to build a cancer subtype classification model, named ERGCN, which performed well on three different TCGA cancer types, presenting a new method for precision cancer treatment.

Therefore, we integrated multi-omics data and designed a model RRGCN based on graph convolution for GC subtype classification. High-dimensional multi-omics data is integrated into low-dimensional space using an artificial neural network autoencoder (AE) to extract hidden layer characteristics. The Patient Similarity Network (PSN) combines the network topology generated by each data type and analyzes the links between patients using the Pearson correlation coefficient (Benesty et al., 2009). The fused network can collect information from multiple data sources that are both shared and complementary. Two residual networks with skip connections are merged with four GCN layers to collect feature matrices and patient similarity correlations to discover and classify GC subtypes, and the classification results are finally output by softmax. The results of the comparison with random forest (RF), support vector machine (SVM), MoGCN, and ERGCN reveal that RRGCN has the best performance. The classification accuracy of the GC subtype is 0.87, the AUC value is 0.98, and the values of other indicators of RRGCN are also the highest when compared to other methods. We believe that RRGCN can provide new and unique insights into the identification, classification, and clinical diagnosis of GC subtypes.

## 2 Materials and methods

### Proposed method

**FIGURE 1:**
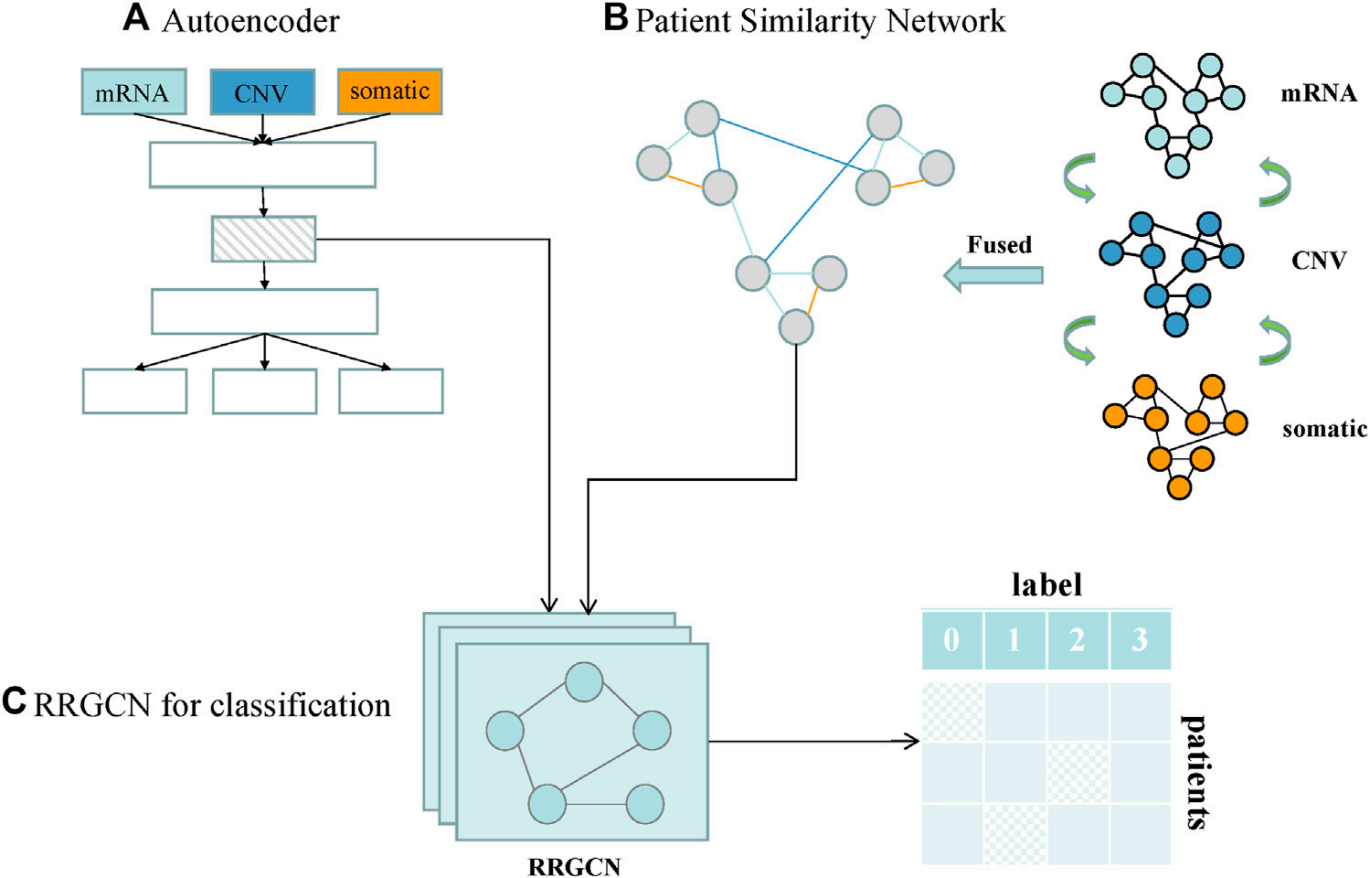
Workflow of RRGCN. **(A)** Features extracted by AE network. **(B)** Patient similarity network constructed by SNF algorithm. **(C)** RRGCN for GC subtype classification.

We designed a GC subtype classification model, namely RRGCN, which is based on the residual graph convolutional network. The input consists of the multi-omics fusion data and the patient similarity network following AE dimensionality reduction. The graph nodes are then embedded through two residual networks with skip connections and a 4-layer GCN, and the classification results are then output using a softmax layer. We compared and assessed RRGCN’s performance with several traditional machine learning models and deep learning methods in the third chapter of the paper. Figure 1 shows the workflow of RRGCN.

### Datasets and data preprocessing

To train the model, we used information on GC from the TCGA (https://tcga-data.nci.nih.gov/tcga/). Transcriptomic data, copy number variations (CNV), and somatic mutation data are all included in our study. We got 272 labeled samples and four subtypes of data from the R tool “TCGAbiolinks” (Colaprico et al., 2016). We download the experimental data using the R package “TCGA-assembler 2” (Wei et al., 2018). The transcriptome data is from the Illumina HiSeq_RNASeqV2 sequencing platform, the CNV data is from the cna_cnv.hg19 sequencing platform and the somatic mutation data is from the somaticMutation_DNAseq sequencing platform. In addition, to make it easier for the model to categorize the input data, we define four GC subtypes as numbers, Epstein-Barr virus type (EBV) as 0, Microsatellite instability type (MSI) as 1, Genetically stable type (GS) as 2, and Chromosome instability type (CIN) as 3.

The dataset in TCGA has to be preprocessed because it contains a large amount of zero and missing value data. The preprocessing step helps to reduce the redundancy and inconsistency of the dataset, thus improving the accuracy and speed of the subsequent mining process. From the phenotypic data, sample information with labels for the various cancer subtypes was first retrieved, and the features that were absent from all samples or had a zero-expression level were subsequently eliminated. So we ended up with 272 samples. Second, among the genes that have been duplicated, we choose the one whose mean expression across all samples has the least absolute value. Finally, for the transcriptome data, we expressed expression levels in units of log2 (FPKM + .1), where FPKM stands for Fragments Per Kilobase of Exon Model per Million mapped Fragment. In this study, we removed the number of zero values and missing values in mRNA, CNV and somatic cells to be 62, 2,481 and 2 respectively, resulting in 20,468, 22,434 and 19,600 features for subsequent model construction. Table 1 shows the details of the dataset.

**TABLE 1:** Overview of the STAD dataset.

Multi-omics	Number of features	Subtypes	Samples
mRNA	20,468	EBV	25
CNV	22,434	MSI	60
Somatic	19,600	GS	51
—	—	CIN	136
Total	62,502	Total	272

### Autoencoder architecture

**FIGURE 2:**
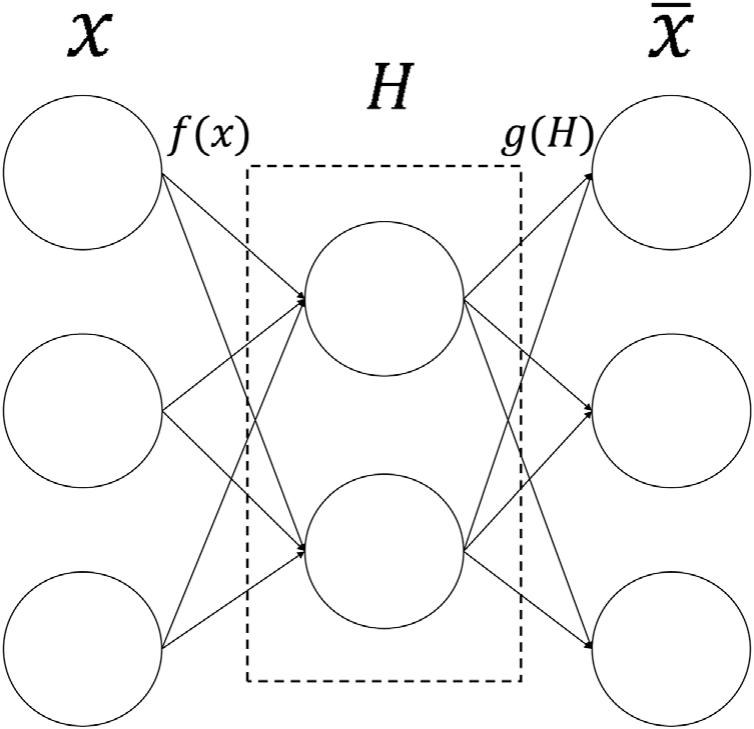
The structure of Autoencoder. The input data is first encoded by the encoder, then embedded in the hidden layer, and finally output by the decoder.

The autoencoder (AE) (Hinton and Salakhutdinov, 2006) is an unsupervised artificial neural network model that belongs to the deep learning category. AE can extract latent embedding representations from multi-omics datasets to reduce dimensionality and computational cost. It can first learn the hidden features of the input data through encoding, then output to the next hidden layer, and then decode and rebuild the original input data with the learned new features (Binbusayyis and Vaiyapuri, 2021). Figure 2 is the basic framework of Autoencoder. The formula is:

$$\begin{array}{l} f\left(x\right)=\delta \left(\omega x+b\right)=H \end{array}$$ (1)

$$\begin{array}{l} g\left(H\right)=\delta \left(\omega^{\prime }H+b^{\prime }\right)=\bar{x} \end{array}$$ (2)

Where x is the input feature in the AE, which is encoded and decoded to $\bar{x}$ . $f\left(x\right)$ represents the encoder function, H represents the hidden unit, $g\left(H\right)$ represents the decoder function, $\bar{x}$ represents the output, δ represents the activation function, ω represents the weight matrix, b represents the bias. We used the mean square error (MSE) (Sammut and Webb, 2010) as the loss function to calculate the loss between the predicted value and the true value, where the predicted value is $\bar{x}$ and the true value is x . The formula is:

$$\begin{array}{l} mseloss\left(x,\bar{x}\right)=\frac{1}{n}\overset{n}{\underset{i=1}{\sum }}{\left(x_{i}-{\bar{x}}_{i}\right)}^{2} \end{array}$$ (3)

Since RRGCN uses three different forms of data, we gave each omics data a varied weight based on prior knowledge (Li et al., 2022) to emphasize their contributions to the model, and all weights sum up to 1. In light of this, the loss function is described as:

$$\begin{array}{l} L_{AE}=a*mseloss\left(x_{1},{\bar{x}}_{1}\right)+b*mseloss\left(x_{2},{\bar{x}}_{2}\right)+c*mseloss\left(x_{3},{\bar{x}}_{3}\right) \end{array}$$ (4)

$L_{AE}$ represents the MSE loss function, and a , b , and c represent the weights of the input data, respectively for 0.4, 0.3, and 0.3. As the input data are characterized by multi-omics data types and represented by multiple matrices $x_{1}$ , $x_{2}$ , and $x_{3}$ , corresponding to the mRNA, CNV, and somatic matrices, ${\bar{x}}_{1}$ , ${\bar{x}}_{2}$ , and ${\bar{x}}_{3}$ correspond to the output of three types of data.

**FIGURE 3:**
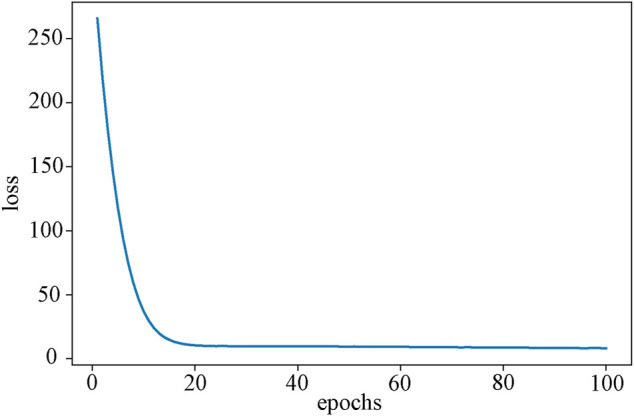
The loss curve of the AE training process. As the epoch grows, training tends to converge around epoch 20.

In this study, we took into account high-dimensional multi-omics data using an AE with three hidden layers. The three hidden layers were (500, 200, 500), and the training epoch was set to 100, which ultimately converged after 20 epochs (Figure 3). All layers employ the sigmoid function as their activation function. AE is trained by back-propagation through the Adam (Kingma and Ba, 2015) optimizer. Additionally, we used grid search to select the batch size from (32, 64, 128) and the learning rate (LR) from (0.01, 0.001, 0.0001). The final batch size is 32, and LR is 0.001. Every model used in our study is built by PyTorch (v1.8.0) (Paszke et al., 2019). The feature matrix extracted by the AE hidden layer will be used as the input of the RRGCN.

### Patient similarity network

The Similarity Network Fusion (SNF) (Wang et al., 2014) algorithm is a computational approach that creates a network of similarities across patients for each type of data to provide a holistic perspective of a certain disease or biological process. We used the SNF algorithm to compute and fuse patient similarity networks from each data type in the GC dataset to create an overall view of GC patients. The advantage of PSN is that it enables RRGCN to seek and obtain important information from the neighbour nodes of the patient, rather than relying solely on the level of gene expression. This improves the accuracy and applicability of the model. The SNF algorithm creates patient-patient similarity matrices for each data type and construct the patient adjacency matrix, then builds a network through the matrix, and lastly fuses various forms of patient-patient similarity networks to create a fusion network. SNF can fully exploit the complementarity of various source data (El-Manzalawy et al., 2018; Picard et al., 2021), which is far superior to the comprehensive analysis approach established by employing a single dataset and has significant advantages in the detection and classification of cancer subtypes (Wang T.-H. et al., 2021; Franco et al., 2021).

Assume there are n samples and m various categories of data (in this study, the data types include mRNA, CNV, and somatic data). We refer to a PSN as a graph $G=\left(V,E\right)$ , where the vertex V is a collection of samples made up of $\left(x_{1},x_{2},\cdots x_{n}\right)$ , and E makes up the edges of the graph. A similarity matrix defined by the scaled exponential similarity kernel was computed:

$$\begin{array}{l} w\left(i,j\right)=\exp\left(-\frac{\rho^{2}\left(x_{i},x_{j}\right)}{\mu \varepsilon_{i,j}}\right) \end{array}$$ (5)

Among them, w represents the similarity matrix between samples, $\rho \left(x_{i},x_{j}\right)$ represents the Euclidean distance between the patient $x_{i}$ and patient $x_{j}$ , μ is a hyperparameter set by experience, and the commonly used range is (0.3, 0.8), and $\varepsilon_{i,j}$ is a parameter used to eliminate the scaling problem, which is defined as:

$$\begin{array}{l} \varepsilon_{i,j}=\frac{mean\left(\rho \left(x_{i},N_{i}\right)\right)+mean\left(\rho \left(x_{j},N_{j}\right)\right)+\rho \left(x_{i},x_{j}\right)}{3} \end{array}$$ (6)

where $N_{i}$ is the set of $x_{i}$ ’s neighbors and $mean\left(\rho \left(x_{i},N_{i}\right)\right)$ is the mean distance from $x_{i}$ to each neighbor. Thus, to compute fusion matrices from multiple data types, the similarity matrix is defined as:

$$\begin{array}{l} P_{i,j}=\left\{\begin{array}{l} \frac{W_{i,j}}{2\sum_{k\neq i}W_{i,k}},\;j\neq i\\ \frac{1}{2},\;j=i \end{array}\right. \end{array}$$ (7)

Then, the affinity matrix S is calculated:

$$\begin{array}{l} S_{i,j}=\left\{\begin{array}{l} \frac{W_{i,j}}{\sum_{k\in N_{i}}W_{i,k}},\;j\in N_{i}\\ 0,\;otherwise \end{array}\right. \end{array}$$ (8)

In the case of various data types:

$$\begin{array}{l} P^{\left(v\right)}=S^{\left(v\right)}\left(\frac{\sum_{k\neq v}P^{\left(k\right)}}{m-1}\right){\left(S^{\left(v\right)}\right)}^{T},v=\mathrm{1,2},\cdots ,m \end{array}$$ (9)

where the $S^{\left(v\right)}$ represents the affinity matrix of *v*th type of data, the $P^{\left(v\right)}$ represents the similarity matrix of *v*th type data. The Pearson correlation coefficient is used to calculate the correlation (linear correlation) between two variables and has a value between −1 and 1. We determined how similar patients were to one another using the Pearson correlation coefficient, and if their similarity exceeded a predetermined threshold, we categorized this as a correlation between patients. The patient similarity network established by the merging of many types of data (multi-omics) is finally obtained by the continual update and iteration of the preceding algorithm.

**FIGURE 4:**
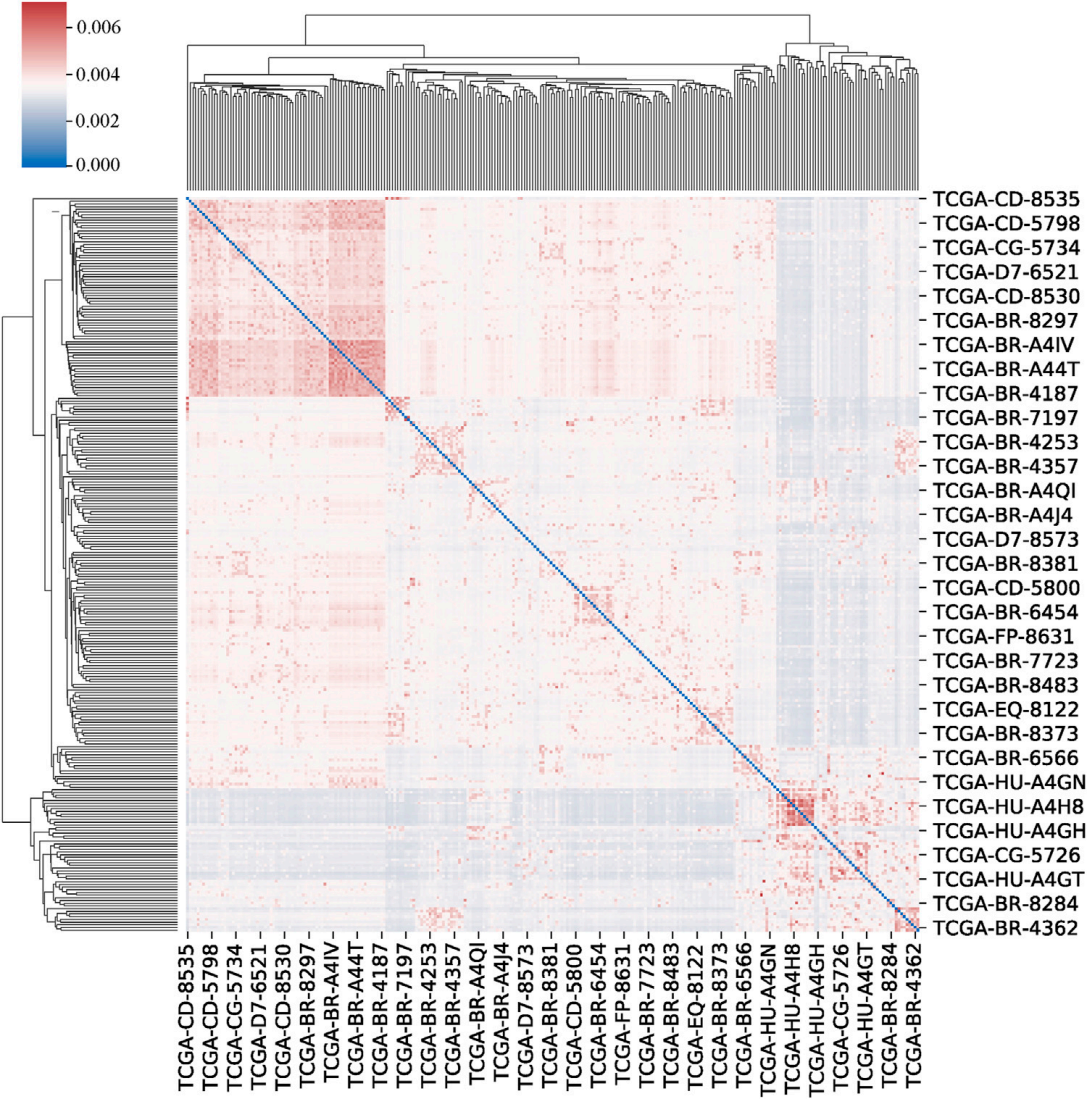
The clustermap of patients. The clustermap shows the clustering relationship among multiple samples, with red representing high correlation and blue representing low correlation.

We set the number of neighbours to consider when creating the affinity matrix to 20 and the scaling factor to 0.5. The clustermap of patients is shown in Figure 4.

### Construction of RRGCN

We use GCN to process non-Euclidean data computed using the SNF algorithm. The purpose of GCN is to learn latent representations based on the node feature matrix X (input, graph nodes) and the similarity matrix A (similarities between nodes). Mathematically, the propagation formula between GCN layers is:

$$\begin{array}{l} H^{L}=\sigma \left({\tilde{D}}^{-\frac{1}{2}}\tilde{A}{\tilde{D}}^{-\frac{1}{2}}H^{L-1}W^{L-1}\right) \end{array}$$ (10)

$H^{L}$ represents the output of the *L*th layer, that is, the node features learned by the *L*th layer, $W^{L-1}$ represents the weight matrix of the L-1-th layer, and σ represents the non-linear activation function in the GCN. D is the degree matrix of A , $\tilde{A}=A+E$ , E represents the identity matrix.

We use the ResNet (He et al., 2016) concept and add skip connections between GCN layers to overcome the problem of model degradation in deep neural network training. The insertion of skip connections can compensate for the loss of features between the data of the previous layer and the data of the following layer, reducing information loss and improving model performance (Yamanaka et al., 2017). At the same time, to avoid the inconsistency between the output of GCN and the dimension of the input data, we add an independent linear layer to the skip connection. The formula for skip connection can be defined as:

$$\begin{array}{l} H^{L+1}=elu\left(H^{L}+linear\left(X\right)\right) \end{array}$$ (11)

$H^{L}$ represents the output of the previous GCN layer. The input feature matrix is sent to the linear layer, and the result is added to the GCN layer and then passed to the non-linear activation function Exponential Linear Units (ELU) (Clevert et al., 2016) to generate the output $H^{L+1}$ , which is utilized as the input of the next skip connection.

**FIGURE 5:**
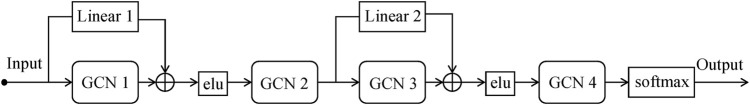
The structure of RRGCN.

RRGCN, which has more skip connections than ERGCN, which only has one, improves model performance by increasing the information flow between layers, making up for information loss, increasing the connectivity between the upper and lower information, and improving the flow of information between layers. The RRGCN as a whole consists of 2 residual networks with skip connections, 4 GCN layers, and 1 softmax layer for generating classification results. To compute the difference between the classification results and the true labels, we utilize the cross-entropy loss function:

$$\begin{array}{l} L=-\left[y\cdot \log\left(\bar{y}\right)+\left(1-y\right)\cdot \log\left(1-\bar{y}\right)\right] \end{array}$$ (12)

where y represents the true label corresponding to the sample, and $\bar{y}$ is the probability value output by the softmax layer. The structure of RRGCN is shown in Figure 5.

**FIGURE 6:**
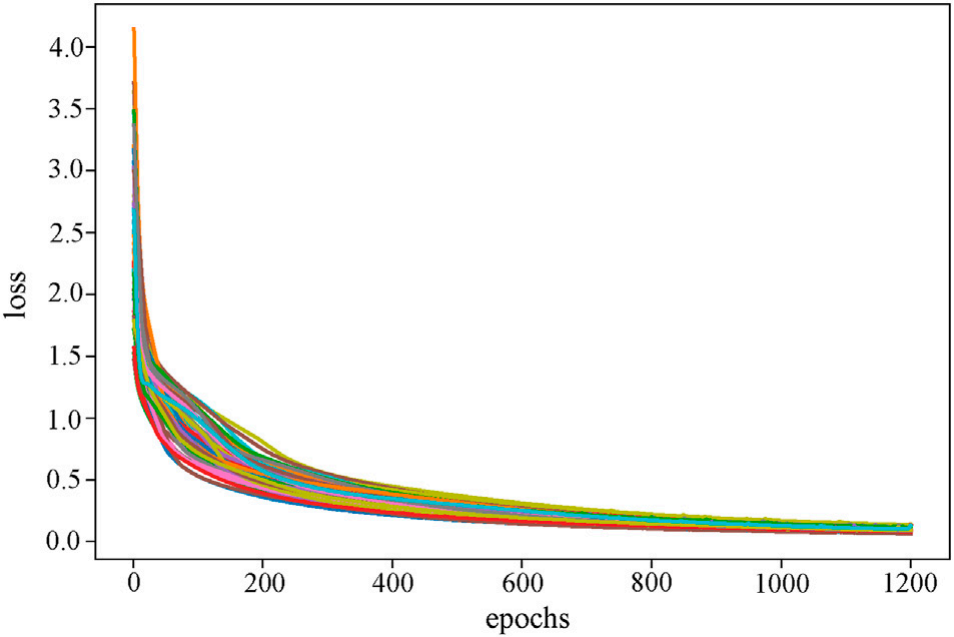
The loss curve of the RRGCN training process. Different colors reflect the loss curves of different cross-validation times.

We set the dimensions of the four graph convolutional layers to 64, 32, 16, and the number of subtypes, respectively. By performing grid search on the LR and weight decay in (0.1, 0.01, 0.001, 0.0001) and (0.1, 0.01, 0.001), respectively, the optimal LR and weight decay are determined to be 0.0001 and 0.01. We use the Adam optimizer function and set the epoch to 1,200, the training process finally converges at 600 (Figure 6). RRGCN employs ELU as the non-linear activation function, and the classification results are finally output *via* the softmax layer. We used 80% of the multi-omics fusion data as the training set and reserved 20% for validation. Model performance was evaluated using 5-fold cross-validation on the training set. Furthermore, to eliminate the bias introduced by a single trial, we took the average outcome of ten iterations of the 5-fold cross-validation test set as the evaluation metric.

### Model evaluation metrics

In the classification task, the model produces four main prediction results: True Positive (TP), False Positive (FP), False Negative (FN), and True Negative (TN). The confusion matrix in Table 2 can be constructed based on the four different prediction outcomes.

**TABLE 2:** Confusion matrix.

Predicted	Actual	
	Positive	Negative
Positive	True Positive (TP)	False Positive (FP)
Negative	False Negative (FN)	True Negative (TN)

Precision refers to the probability that the prediction is correct in the sample that is predicted to be true. It is defined as:

$$\begin{array}{l} precision=\frac{TP}{TP+FP} \end{array}$$ (13)

Recall, also known as sensitivity, is the measure of how many samples are selected as being true. It is defined as:

$$\begin{array}{l} recall=sensitivity=\frac{TP}{P} \end{array}$$ (14)

The F1 score is a weighted harmonic average of precision and recall that is unaffected by imbalanced samples. The F1 score has a maximum value of one and a minimum value of zero. The higher the value, the higher the model quality. In most circumstances, the f1 score can be used directly to evaluate and pick the model, and some well-known machine learning competitions do as well. It is defined as:

$$\begin{array}{l} F1\;score=\frac{2*precision*recall}{precision+recall} \end{array}$$ (15)

Accuracy is defined as the ratio of accurately predicted samples to total samples. It is defined as:

$$\begin{array}{l} Accuracy=\frac{TP+TN}{TP+TN+FP+FN} \end{array}$$ (16)

The area contained by the curve with the false positive rate (FPR) on the abscissa and the true positive rate (TPR) on the ordinate is known as the area under the receiver operating characteristic curve (ROC) curve (AUC). The categorization skill given by the ROC curve is intuitively reflected by AUC. The AUC value ranges between 0 and 1, and the higher the value, the better the classifier’s performance. FPR is the likelihood that the prediction is a positive sample but the prediction is incorrect. It is defined as:

$$\begin{array}{l} FPR=\frac{FP}{TN+FP} \end{array}$$ (17)

TPR reflects the likelihood that the forecast is a positive sample and that the prediction is right. It is defined as:

$$\begin{array}{l} TPR=\frac{TP}{TP+FN} \end{array}$$ (18)

The area contained by the curve with recall on the abscissa and precision on the ordinate is known as PR-AUC, and it is the mean value of precision calculated for each recall threshold (Géron, 2017). All model evaluation metrics are based on Scikit-learn (Pedregosa et al., 2011).

## 3 Results

### Determination of pearson correlation threshold

**FIGURE 7:**
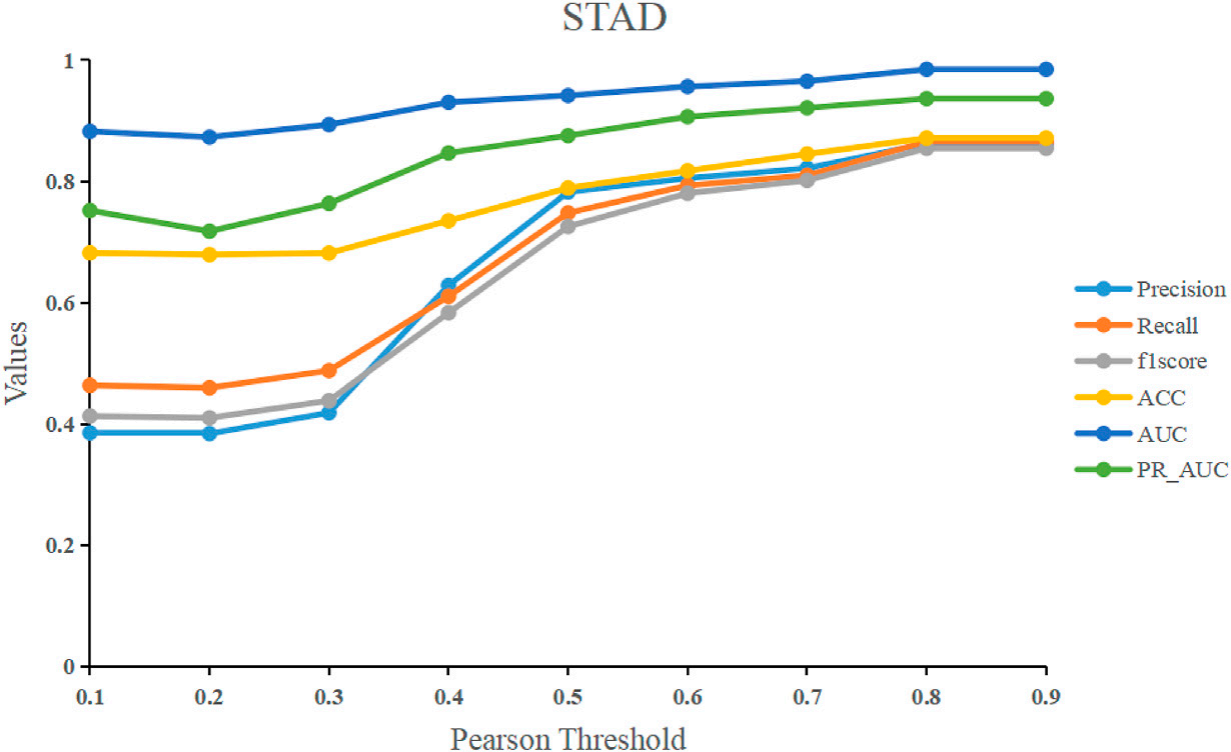
The effect of different Pearson correlation thresholds on model performance.

We used the Pearson correlation threshold to see if there was a link between samples. If the Pearson correlation coefficient between samples is larger than the threshold, we connect the two samples with an edge and set the corresponding value in the adjacency matrix to 1. In contrast, there is no edge connecting the two samples, and the corresponding values in the adjacency matrix are 0. To examine the performance of the models, we fixed the threshold to a value ranging from 0.1 to 0.9. Figure 7 shows that before 0.5, the model’s performance improves significantly as the threshold is raised. After 0.5, it tends to be flat, and the model’s performance peaks at the final threshold of 0.8. Our model RRGCN performed best when Pearson correlation threshold was 0.8, where the Precision, Recall, F1score, ACC, AUC and PR_AUC reached 0.862, 0.865, 0.854, 0.871, 0.984, and 0.936, respectively.

### Performance of RRGCN in multi-omics

To verify the superiority of multi-omics data, as well as the validity and contribution of each type of data to the model, we conduct experiments on different types of data separately. From the experimental results (Table 3), it can be seen that using a single omics data training model, the highest performance is the mRNA group with an accuracy of 0.7384, followed by the somatic group with an accuracy of 0.5190. The CNV group has the lowest accuracy, only 0.4766. It can be seen that although RNA-seq data has good performance and is indeed used in most studies, its effect is still inferior to multi-omics data. Of course, this also reflects from a certain level that RNA-seq data contains extremely important biological information, has good performance in the classification of cancer subtypes, and is extremely important for cancer diagnosis and treatment (Yang et al., 2021). It can be verified that there is complementary information between different omics, which can explain the nature of cancer from different perspectives and improve the diagnostic efficiency of cancer.

**TABLE 3:** Results of multi-omics data compared with single-dimensional data.

Omics	Accuracy	AUC
mRNA	0.7384	0.9339
CNV	0.4766	0.7809
Somatic	0.5190	0.8272
Multi-omics	**0.8713**	**0.9848**

Bold values emphasize that the experimental results of multi-omics are better than other groupings.

### Comparison with other classical methods

To validate RRGCN’s classification performance, we compare it to two other classical machine learning methods and two graph convolution-based classification approaches and evaluate it using four standard external evaluation measures. We employ four classification methods: Random Forest (Breiman, 2001), Support Vector Machine (Cortes and Vapnik, 1995), MoGCN (Li X. et al., 2022), and ERGCN (Dai et al., 2021).

·Random Forest (RF) is essentially a bagging algorithm, which randomly selects a feature from the most important features for branching, creates multiple decision trees, and finally votes on which category the data finally belongs to.
·Support Vector Machine (SVM), a binary classification model whose basic model is defined as a linear classifier with the biggest margin on the feature space. The goal is to build an objective function based on the structural risk reduction principle that distinguishes between the two types as much as possible.
·MoGCN is a multi-omics integration model based on GCN. The model utilizes feature extraction and network visualization for further biological knowledge discovery and subtype classification.
·ERGCN is a cancer subtype classification method based on residual graph convolutional networks and sample similarity networks for gene co-expression patterns.

To begin, we unified the AE latent layer feature matrix as input data for each model to ensure the rigor of the compared tests. Then, we utilized scikit-learn to construct these algorithms and grid search to optimize the RF and SVM parameters. The best number of sub-decision trees (n_esimators) for RF is between 1 and 101, with a step size of 10. Finally, 5-fold cross-validation yielded an optimal n_esimators of 26. The maximum number of features (max_features) should ideally be between 1 and 21, with a stride of 1. Finally, the optimal max_features is selected as 20 through 5-fold cross-validation. We also use grid search for SVM, choosing the penalty coefficient (C) from (0.1, 1, 100, 1,000) and the kernel function coefficient (gamma) from (0.0001, 0.001, 0.005, 0.1, 1, 3, 5), as well as the kernel function (kernel) from (“linear,” “rbf”). The final optimized C is 1,000, the gamma is 0.001, and the kernel is “rbf.” For MoGCN and ERGCN, we use the optimal parameters already set by their authors. The model comparison results are shown in Table 4.

**TABLE 4:** Results in comparison to other methods.

Model	Accuracy	F1 score	Precision	Recall
RF	0.8363	0.7665	0.8172	0.7471
SVM	0.7455	0.7340	0.7792	0.7460
MoGCN	0.7944	0.8078	0.8034	0.7407
ERGCN	0.7901	0.6826	0.7160	0.6120
RRGCN	**0.8713**	**0.8544**	**0.8621**	**0.8654**

Bold values are to highlight the performance of our model over other classical models.

From the results, we can see that RRGCN has an excellent performance in the classification of GC subtypes. The classification accuracy of RRGCN is as high as 0.8713, which is 5.49% higher than the best RF among the other four methods, and 11.47%, 8.83%, and 9.32% higher than the other three methods, respectively. The F1 score, Precision, and Recall of RRGCN are 0.8544, 0.8621, and 0.8654 respectively, and the values are also much higher than other methods. Most crucially, as compared to ERGCN, RRGCN performs better on each of the four evaluation metrics by 10.28%, 25.17%, 20.41%, and 41.41%, respectively. In defining the various subtypes of GC, RRGCN has more advantages. In the future, it might be used to treat more diseases, offering novel perspectives on how to diagnose and treat clinical illnesses.

## 4 Discussion

Heterogeneity causes cancer to differentiate into different subtypes, and subtypes with different degrees of differentiation and malignancy have different sensitivities to clinical therapeutic drugs, which brings great challenges to the diagnosis and treatment of the disease (Lin et al., 2021; Yuan et al., 2022). GC is a highly heterogeneous tumor, and its average somatic gene copy number changes are much higher than those of other tumor types (Joshi and Badgwell, 2021). Therefore, in clinical studies, the progression of GC is the slowest (Li et al., 2021).

Therefore, by integrating multi-omics data, we propose a graph convolutional network based on residual networks to realize the subtype classification of GC. Multi-omics datasets are dimensionally reduced by AE to extract representative latent layer features. The SNF algorithm is used to find the associations existing between patients. Finally, PSN combined with the feature matrix was input into RRGCN, and the classification results were output through the softmax layer. The results show that the accuracy of RRGCN reaches 0.8713.

The improvement of RRGCN over previous models is that multi-omics data is used as the basis of research, and the neglected similarity between patients is combined as the input of the model. For model selection, we introduce two skip connections to alleviate the loss of information during training and solve the model degradation problem.

To explain the advantage of multi-omics data, we retrain three different types of data separately and compare the results with multi-omics data. The results show that the performance of the model trained with multi-omics data is much higher than that of the single-omics data, and the accuracy is improved by about 18.00%. To prove the superiority of RRGCN, we compare RRGCN with classical machine learning methods and well-performing deep learning models, respectively. The results show that the performance of RRGCN is higher than other methods in all aspects. Most importantly, the accuracy of RRGCN is 10.28% higher than that of ERGCN.

The model is sensitive to the selection of the Pearson threshold, and the supervised learning method also brings inconvenience to the selection of data. In the future, we will focus on studying the application of graph convolution combined with other classical convolutional neural networks, considering the development of new unsupervised learning methods for cancer subtype recognition and classification.

## 5 Conclusion

In summary, we proposed a new classification method for gastric cancer subtypes called RRGCN by borrowing skip connections in residual networks. Through the deep mining of GC multi-omics data and the consideration of the relationship between patients, and comparing RRGCN with other classical machine learning methods and deep learning models, we verify the excellent performance of RRGCN in various aspects and improve the cancer subtype classification method to a higher level. The development of new models opens up new avenues for precise treatment. Li J. et al., (2022), Yang et al., (2022), and Hu et al., (2021). have tried to combine GCN with spatial transcriptomics for cell clustering and the identification of cancer subtypes. In the future, we will look into the spatial coordinate information of gastric cancer cells and employ unsupervised learning algorithms to provide more robust support for clinical diagnosis and treatment of gastric cancer.

## Data Availability

The original contributions presented in the study are included in the article/supplementary material, further inquiries can be directed to the corresponding author.
